# Comparative effects of selenium-enriched lactobacilli and selenium-enriched yeast on performance, egg selenium enrichment, antioxidant capacity, and ileal microbiota in laying hens

**DOI:** 10.1186/s40104-025-01160-6

**Published:** 2025-02-19

**Authors:** Jianmin Zhou, Uchechukwu Edna Obianwuna, Longfei Zhang, Yongli Liu, Haijun Zhang, Kai Qiu, Jing Wang, Guanghai Qi, Shugeng Wu

**Affiliations:** 1 Key Laboratory of Feed Biotechnology, Ministry of Agriculture and Rural Affairs, Institute of Feed Research, China Academy of Agricultural Sciences, Beijing, 100081 People’s Republic of China; 2 College of Animal Science and Technology, Beijing Agricultural University, Beijing, 100096 China; 3 Baiyian Biological Engineering Co., Ltd., Jiaozuo, Henan 454000 China

**Keywords:** Antioxidant capacity, Egg selenium, Feed efficiency, Gut microbiota, Laying hen, Selenium-enriched lactobacilli, Selenium-enriched yeast

## Abstract

**Background:**

Organic selenium (Se) has gained recognition in poultry nutrition as a feed additive to boost production and Se deposition in eggs and tissues, owing to its high bioavailability, efficient tissue accumulation and minimal toxicity. Selenium-enriched yeast (SeY) is a well-established source, while selenium-enriched lactobacilli (SeL), a newer alternative, offers the added benefits of probiotics. This study examined the effects of SeY and SeL on egg quality, antioxidant capacity, Se deposition, and gut health in laying hens. After a two-week pre-treatment with a Se-deficient diet (SeD), 450 Hy-Line Brown laying hens (30-week-old) were assigned into five dietary groups with six replicates of 15 hens each. The groups included a SeD, SeD supplemented with 1.5 mg Se/kg from SeY (SeY15), or 1.5, 3.0, and 6.0 mg Se/kg from SeL (SeL15, SeL30, SeL60). The feeding trial lasted for 12 weeks.

**Results:**

SeY15 and SeL15 improved the feed-to-egg ratio (*P* < 0.05) in the latter stages. Haugh units were significantly increased (*P* < 0.05) in the SeY15 and SeL30 groups, while darker yolk color (*P* < 0.05) was observed in the SeY15, SeL15, and SeL60 groups. All Se-supplemented diets increased Se content in whole eggs, albumen, and yolk (*P* < 0.05), while SeL groups showed a dose-dependent effect. Antioxidant enzyme activities increased, and MDA content decreased in the serum (*P* < 0.05), with SeY15 showing the highest GSH-Px levels (*P* < 0.05). SeL60 increased serum alkaline phosphatase and aspartate transaminase, and distorted the liver architecture (*P* < 0.05). Se-diets reduced concentrations of reactive oxygen species (ROS) in the ileum and liver (*P* < 0.05). SeL15 improved the ileal villus height-to-crypt depth ratio (*P* < 0.05). SeY15 and/or SeL15 up-regulated *TXNRD1* and *SEPHS1* mRNA while down-regulating *SCLY* expression in the liver. SeY15 altered ileal microbiota by increasing both beneficial and pathogenic bacteria, whereas SeL15 predominantly boosted beneficial bacteria.

**Conclusion:**

SeL integrates the antioxidant properties of organic Se with the probiotic benefits on gut health, resulting in a performance-enhancing effect comparable to that of SeY. However, high SeL level (6.0 mg Se/kg) compromised productivity and metabolic functions while enhancing Se deposition.

## Introduction

Selenium (Se) is an essential micro-element for both animals and humans. As an integral component of several enzymes, including glutathione peroxidase (GSH-Px), type I iodothyronine deiodinase, and thioredoxin reductase, Se plays crucial roles in various metabolic functions such as preventing oxidative stress, regulating thyroid hormones, maintaining cellular redox balance, and supporting immunocompetence [1–3]. Furthermore, Se contributes to the detoxification of heavy metals and xenobiotics [4], highlighting its importance in metabolic health. Given these essential functions, Se deficiency is a significant global health concern, affecting nearly one billion individuals worldwide [5]. In China, approximately 72% of the county’s population is Se-deficient [6], with some regions reporting daily Se intake as low as 10 μg [7], far below the Chinese Dietary Reference Intakes recommendation of 50–60 μg/d for adults. This deficiency has been linked to the endemic occurrence of Keshan disease and Kaschin-Beck disease [8]. Similarly, in poultry production, Se deficiency in laying hens can lead to decreased productivity in laying hens, resulting in significant economic losses [9].

Se fortification of food products is widely regarded as an effective strategy to mitigate Se deficiency [2]. In the poultry industry, there has been a growing emphasis on incorporating elevated doses of Se into chicken feed, aiming to enhance the Se content in carcass meat and eggs [10–14]. Traditionally, sodium selenite has been the most common Se supplement in poultry feed, but organic Se forms are also less toxic and environmentally more damaging compared to inorganic forms, making them a preferred choice for sustainable poultry production [15]. Among the organic sources of Se, selenomethionine (Se-Met) and selenium-enriched yeast (SeY) have been approved as Se feed additives in animal nutrition [16–18]. Also, Se-Met and SeY have gained prominence due to their superior bioavailability and enhanced antioxidant properties [3, 14]. For instance, SeY is primarily composed of Se-Met, which can be incorporated directly and non-specifically into proteins (e.g., carcass meat, eggs, and milk) instead of methionine [19], making it more valuable in the food chain.

The European Food Safety Authority [20] reported that the inclusion of SeY at 0.5 mg Se/kg in layer diets significantly increased (~50%) the Se content of eggs compared to sodium selenite supplementation at the same dose, and no adverse effects were observed with higher SeY supplementation levels of up to 5.7 mg Se/kg. Additionally, other organic Se sources such as 2-hydroxy-4-methyselenobutanoic acid [21], Se-enriched kale sprout [22], Se-enriched alga [23], selenized glucose [13], Se-polysaccharides [14, 24], and Se-enriched probiotics [25, 26], have also been explored in poultry nutrition.

Se-enriched lactobacilli (SeL) employed a specific strain of lactobacilli that is abundant in organic Se-containing proteins, serving as a biotransformation vector to convert inorganic Se into organic forms. SeL may combine the benefits of organic Se with probiotic properties, offer new opportunities for enhancing both Se absorption and gut health. Previous studies have shown that application of Se-enriched probiotics (*Lactobacillus* spp.), such as selenium- and zinc-enriched *Lactobacillus plantarum* in mouse model [27], selenium-enriched *Lactobacillus acidophilus* [28], and selenium nanoparticles-enriched *Lactobacillus casei* ATCC 393 P [29] in mice. All of these highlight the mitigative effect of Se on oxidative stress, via regulatory effect on immune and inflammatory response, antioxidant function, and gut microbiota composition. Despite its potentials, research on the effects of SeL on laying hens remains largely unexplored, particularly regarding its impact on production performance, egg quality, Se concentration in eggs, antioxidant capacity, and ileal microbiota. Additionally, it is critical to evaluate the tolerance of hens to SeL, as Se has a narrow therapeutic window, with excessive intake potentially leading to toxicity [30].

In this study, we investigated the biological effects of SeL on production performance, egg quality, Se concentration in eggs, antioxidant capacity, and ileal microbiota in laying hens. We used SeY to serve as the reference, due to its established efficacy and wide applicability. Furthermore, we evaluated the safety of high-dose SeL by analyzing serum biochemical parameters and liver histomorphology for signs of toxicity. These findings aim to contribute to the optimization of organic Se supplementation strategies in poultry production, providing insights into the potential benefits of probiotics for enhanced Se utilization.

## Materials and methods

### Ethic statement

Animal protocols in this study were approved by the management of the Animal Care and Use Committee of Institute of Feed Research, Chinese Academy of Agricultural Sciences (approval No. AEC-CAAS-20230622).

### Animal management and experimental design

A total of 450 30-week-old Hy-line Brown laying hens were allocated into one of five treatments according to the completely randomized design, with 6 replicates of 15 birds each. Laying hens were allocated to 3-tier battery cages of 3 birds each (cage size: 40 cm × 40 cm × 35 cm) and exposed to 16 h of light/d with an intensity of 20 lx. Experimental hens had similar initial body weight and laying rate across all the replicates. Diets and water were offered ad libitum in mash form and by nipple drinkers, respectively. The experiment lasted for 14 weeks, including a 14-d preliminary feeding period and an 84-d experimental feeding period. All hens remained in good health during the feeding period.

During the preliminary feeding period, all birds were fed with a Se-deficient (SeD) basal diet to consume the body’s stores of Se. The corn-soybean meal basal diet formulated according to the Chinese Feeding Standard of Chickens (NY/T33-2004) [31] is shown in Table 1. After Se-depletion, one group was still offered SeD diet, while the remaining 4 groups were supplemented with SeY or SeL to bring the total Se contents to 1.5 (SeY15), 1.5 (SeL15), 3.0 (SeL30), and 6.0 (SeL60) mg/kg, respectively. The Se-enriched yeast and Se-enriched lactobacilli were respectively purchased from Yinglian Food Additives Co., Ltd. (Harbin, China) and Baiyian Bioengineering Co., Ltd. (Jiaozuo, China). Diet samples (approximately 250 g each) were collected with quartering division method for nutrients analyses. The analyzed values of Se contents are shown in Table 2.

**Table 1:** Composition and nutrient levels of the basal diet (as-fed basis)

Ingredient	Content, %
Corn	64.67
Soybean meal (44.8% CP)	23.50
Soybean oil	0.60
Lime stone	9.00
Dicalcium phosphorate	0.84
Sodium chloride	0.15
Sodium bicarbonate	0.65
DL-Methionine (98%)	0.17
L-Lysine-HCl (98%)	0.02
L-Threonine (98%)	0.04
Choline chloride (50%)	0.20
Premix^a^	0.13
Phytase	0.03
Total	100
Nutrient level^b^	
Metabolizable energy, kcal/kg	2,709
Crude protein	16.07 (16.45)
Calcium	3.50 (4.35)
Total phosphorus	0.53 (0.45)
Nonphytate phosphorus	0.32
Lysine	0.75 (0.751)
Methionine	0.39 (0.405)
Methionine + cysteine	0.65 (0.685)
Threonine	0.55 (0.613)
Selenium	0 (0.042)

^a^Premix supplied per kilogram of diet: vitamin A, 12,500 IU; vitamin D_3_, 4,125 IU; vitamin E, 15 IU; vitamin K_3_, 2 mg; thiamine, 1 mg; riboflavin, 8.5 mg; pyridoxine, 8 mg; vitamin B_12_, 0.04 mg; biotin, 0.1 mg; folic acid, 1.25 mg; Ca-pantothenate, 50 mg; niacin, 32.5 mg; Cu, 8 mg; Zn, 80 mg; Fe, 40 mg; Mn, 90 mg; I, 1.2 mg. Selenium in each treatment group is shown in the experiment design in Table 2

^b^The values in parenthesis indicate analyzed values. Others are calculated values

**Table 2:** The Se level of experimental diets

Item	Experimental treatment^a^				
	SeD	SeY15	SeL15	SeL30	SeL60
Measured value, mg/kg	0.042	1.773	1.681	3.378	6.061

^a^*SeD* The basal diet without Se supplementation, *SeY15* The diet supplemented with 1.5 mg/kg Se from Se-enriched yeast, *SeL15*, *SeL30* or *SeL60* The diets supplemented with 1.5, 3.0 or 6.0 mg/kg Se from Se-enriched lactobacilli

### Data and sample collection

At the end of the trial, eight birds (including one per replicate and two additional birds randomly selected from each group) from each of SeD, SeY15, SeL15, and SeL60 treatments for sample collection. Blood samples were taken from the wing vein, incubated in a 37 °C water bath for 10 min, and centrifuged at 1,000 × *g* for 15 min to harvest serum. The serum samples were stored at −20 °C until analysis. Subsequently, the selected birds were slaughtered and dissected under aseptic conditions. Thigh muscles were collected from the left side of the birds for Se content analysis. Two random pieces of fresh liver and two segments (approximately 2-cm long) of the middle portion of the ileum were taken, one of each tissue sample was fixed in 4% paraformaldehyde for histomorphology analysis, while the other was frozen in liquid nitrogen, and stored at −80 °C for reactive oxygen species (ROS) immunofluorescence assay. Liver tissues and ileal contents were collected in sterile containers, frozen in liquid nitrogen, followed by storage at −80 °C until analysis.

### Laying performance and egg quality

Mortality was recorded as it occurred. Daily egg number, total egg weight and biweekly feed consumption were recorded. Hen-day egg production, egg mass, average egg weight, average daily feed intake, and feed-to-egg ratio were calculated out based on the periods of weeks 1–6, weeks 7–12, and weeks 1–12. Feed-to-egg ratio was calculated as grams of feed consumed per grams of eggs produced.

Six eggs from each replicate with the weight close to replicate average were collected at the start of experimental feeding period (week 0) and the end of weeks 6 and 12. Eggshell thickness (blunt end, tip, and equator) and the eggshell strength were measured by an Eggshell Thickness Gauge and an Egg Force Reader (ORKA Technology Ltd., Ramat HaSharon, Israel), respectively. Albumen height, Haugh unit (HU), and yolk color were measured using the Egg Analyzer™ (ORKA Food Technology Ltd., Ramat HaSharon, Israel).

### Se contents in egg and thigh muscle

At the end of week 12, 6 eggs/replicate were randomly selected and broken. Three whole liquid eggs (also called edible egg part) or albumens/yolks of which were pooled as one sample for Se content evaluate. Based on the China National Standard GB 5009.93–2017 [32], the Se contents in experimental diets, whole liquid eggs, albumens/yolks and thigh muscles were determined by hydride atomic fluorescence spectrometry (iCE 3300 AAS, Thermo Fisher Science, Rockford, IL, USA). Certified reference material (chicken muscle, GBW10018) was used for quality control in Se detection [33].

### Serum parameters

Serum alkaline phosphatase (ALP), aspartate transaminase (AST), alanine transaminase and uric acid were determined using automatic biochemical analyzer (Zhuoyue 300, Kehua Bio-engineering Co., Ltd., Shanghai, China). The measurement of serum antioxidant indices inclusive of total antioxidant capacity (T-AOC), catalase (CAT), GSH-Px, superoxide dismutase (SOD), and malondialdehyde (MDA) was performed by assay kits for chickens (Shanghai Enzyme-linked Biotechnology Co., Ltd., Shanghai, China) following the manufacturer’s instructions.

### Histomorphology of liver and ileum

Liver pieces and ileal segments were washed, dehydrated, clarified, and embedded in paraffin. Sections were cut at 5-micron thickness, fixed with neutral balsam and stained with hematoxylin and eosin (H&E), and then inspected under a light microscopy. Three intact villi-crypt units were randomly selected for ileal morphometric evaluation, including villus height (VH, the height from the tip of the villus to the villus-crypt junction), crypt depth (CD, the depth from the base up to the crypt-villus transition region) and the villus height-to-crypt depth ratio (VH/CD).

### ROS fluorescence staining in liver and ileum tissues

Dihydroethidium fluorescence staining is a simple method for ROS detection in tissues or cells [34]. Fresh-frozen liver and ileum samples were serially sectioned at 10-micron thick sections using a Leica CM1900 cryostat (Shanghai, China). Sections were incubated at 37 °C for 60 min with 10 μmol/L DHE in the dark, counterstained with the nuclear stain 4,6-diamidino-2-phenylindole dichlorohydrate (DAPI, 300 nmol/L), and in the following 24 h inspected under an Olympus DP26 fluorescence microscope (Tokyo, Japan). The level of ROS was expressed in relative fluorescence intensity which was calculated using Image-Pro Plus software.

### RNA isolation and real-time quantitative PCR

Total RNA of liver was extracted using EasyPure RNA kit (TransGen Biotech Co., Ltd., Beijing, China). RNA concentration was determined using an Epoch Microplate Spectrophotometer (BioTek Instruments, Inc., VT, USA). Reverse transcription reactions were performed using the First-Strand cDNA Synthesis SuperMix (TransGen Biotech Co., Ltd., Beijing, China). Quantitative PCR assays were conducted in triplicate in a CFX96 C1000^TM^ thermal cycler (Bio-Rad Laboratories, CA, USA). The relative gene expression levels were calculated using the 2^−ΔΔCt^ method [35] and the primer sequences are listed in Table 3.

**Table 3:** Gene-specific primers for real-time quantitative reverse transcription PCR

Gene^a^	Primers (5′→3′)	Accession No.
*SCLY*	Forward: GGGACACCTGGCAGAAACTACC	NM_001139463.1
	Reverse: CGCACCGCAGCAAGAATATCATC	
*TXNRD1*	Forward: TATGGAGCCTGCGGATATTCTGAAG	NM_001030762.4
	Reverse: AGCATTTGTTGTTGTCTCTGGATGG	
*TXNRD3*	Forward: GATGTCACAGTAATGGTGCGTTCC	NM_001122777.3
	Reverse: GTGCCATCCTCCAGCCTTTCC	
*SEPHS1*	Forward: GCCAAGCAGCAGCGAAATGAG	NM_001164084.4
	Reverse: AGCCTTACTGACAGCAGCCATC	
*GPX1*	Forward: GTGCGAGGTGAACGGGAAGG	NM_001277853.3
	Reverse: AGATGATGTACTGCGGGTTGGTC	
*GPX2*	Forward: GAGAACGGCACCAACGAGGAG	NM_001277854.3
	Reverse: TTCACCTGGCACTTCTGGAACAG	
β-actin	Forward: TATGTGCAAGGCCGGTTTC	NM_205518.2
	Reverse: TGTCTTTCTGGCCCATACCAA	

^a^*SCLY* Selenocysteine lyase, *TXNRD1* Thioredoxin reductase 1, *TXNRD3* Thioredoxin reductase 3, *SEPHS1 S*elenophosphate synthetase 1, *GPX1 G*lutathione peroxidase 1, *GPX2* Glutathione peroxidase 2

### 16S rRNA sequencing of ileal microbiota

Total DNA was extracted from frozen ileal content samples using the E.Z.N.A.® Soil DNA Kit (Omega Bio-tek, Norcross, GA, USA) according to manufacturer’s instructions. The quality of DNA samples was assessed by gel electrophoresis. The hypervariable regions V3–V4 of the bacterial 16S rRNA genes were amplified with primer pairs 338F/806R (5′-ACT CCT ACG GGA GGC AGC AG-3′ and 5′-GGA CTA CHV GGG TWT CTA AT-3′). The PCR amplification was performed as follows: initial denaturation for 3 min at 95 °C, followed by 27 cycles of denaturation at 95 °C for 30 s, annealing at 55 °C for 30 s and extension at 72 °C for 45 s, with a final extension at 72 °C for 10 min. PCR products were extracted from a 2% agarose gel and purified using the AxyPrep DNA Gel Extraction Kit (Axygen Biosciences, Union City, CA, USA) according to the manufacturer’s instructions. Purified amplicons were qualified and paired-end sequenced on Illumina MiSeq PE300 platform (Illumina, San Diego, USA) according to the standard protocols by Majorbio Bio-pharm Technology Co., Ltd. (Shanghai, China). The row reads were deposited into NCBI Sequence Read Archive (SRA) database (Accession Number: PRJNA1120179).

### Statistical analysis

Data other than ileal microbiota were analyzed by one-way Analysis of Variance (ANOVA) procedure and means were compared using Duncan’s Multiple Range Test with SAS 9.2 (SAS Institute Inc., Cary, NC, USA). The linear and quadratic effects of SeL addition levels on egg Se concentration were assessed using regression analysis. Differences were considered statistically significant at *P* < 0.05. Data are expressed as means and their pooled standard error of the mean (SEM).

Microbial data analysis was conducted on the free online platform of Majorbio Cloud Platform (www.majorbio.com) of Shanghai Majorbio Bio-pharm Technology Co., Ltd. The sequencing results were analyzed based on amplicon sequence variants. Alpha diversity metrics were employed to assess microbial richness and evenness, including ACE, Chao, Shannon and Simpson indices. Beta diversity was established by principal coordinate analysis (PCoA) based on Bray–Curtis distance. The significance of differentiation among microbial profiles of treatments was assessed by analysis of similarity (ANOSIM). Linear discriminant analysis (LDA) combined effect size measurements (LEfSe) were employed using all-against-all strategy. The LDA was used to estimate the effect size of each differentially abundant feature, and the threshold on the LDA score (log_10_LDA) was set as 3.0. Wilcoxon rank-sum test was performed to explore general differences between groups.

## Results

### Laying performance and egg quality

As shown in Table 4, no differences in laying performance were observed during the first 6 weeks. However, during 7–12 weeks, dietary Se at a high level of 6.0 mg Se/kg diet resulted in a reduction in hen-day egg production and average daily feed intake (*P* < 0.05). In contrast, birds in the SeY15 and SeL15 groups showed a more favorable feed-to-egg ratio compared to the SeD group (*P* < 0.05). Throughout the entire experimental period, hen-day egg production and egg mass were significantly higher in the SeY15 and SeL15 groups than in the SeL60 group (*P* < 0.05).

**Table 4:** Effect of dietary Se-enriched lactobacilli on performance of laying hens^1^

Item	Experimental treatment^2^					SEM	*P* value
	SeD	SeY15	SeL15	SeL30	SeL60		
Egg production, %							
1–6 weeks	88.29	89.83	90.68	88.38	86.42	0.825	0.562
77–12 weeks	83.17^a^	84.36^a^	86.21^a^	78.62^ab^	76.99^b^	0.901	0.001
1–12 weeks	85.73^ab^	87.10^a^	88.45^a^	83.50^ab^	81.70^b^	0.800	0.042
Average egg weight, g							
1–6 weeks	56.94	57.33	56.43	57.18	58.39	0.239	0.110
7–12 weeks	57.30	58.12	57.66	57.47	59.38	0.286	0.136
1–12 weeks	57.12	57.72	57.04	57.33	58.88	0.256	0.131
Average daily feed intake, g							
1–6 weeks	104.25	104.92	105.49	104.17	103.17	0.566	0.781
7–12 weeks	110.23^a^	109.36^ab^	108.69^ab^	103.56^b^	104.61^b^	0.869	0.035
1–12 weeks	107.24	107.14	107.09	103.86	103.89	0.627	0.154
Egg mass, g							
1–6 weeks	50.27	51.51	51.18	50.55	50.46	0.513	0.944
7–12 weeks	47.65^ab^	49.03^a^	49.72^a^	46.03^b^	45.57^b^	0.471	0.008
1–12 weeks	48.96	50.28	50.47	47.88	48.12	0.499	0.344
Feed-to-egg ratio, g/g							
1–6 weeks	2.08	2.04	2.06	2.07	2.06	0.020	0.988
7–12 weeks	2.20^a^	2.12^bc^	2.08^c^	2.15^ab^	2.18^ab^	0.013	0.007
1–12 weeks	2.14	2.08	2.07	2.12	2.12	0.013	0.424

^a−c^Within a row, means with no common superscript differ significantly (*P* < 0.05)

^1^Means were calculated using 6 replicates per treatment (15 birds of each replicate)

^2^*SeD* The basal diet without Se supplementation, *SeY15* The diet supplemented with 1.5 mg/kg Se from Se-enriched yeast, *SeL15*, *SeL30* or *SeL60* The diets supplemented with 1.5, 3.0 or 6.0 mg/kg Se from Se-enriched lactobacilli

The effects of dietary treatments on egg quality are summarized in Table 5. Both the SeY15 and SeL30 treatments significantly improved HU at the conclusion of the trial (*P* < 0.05). Additionally, SeY15, SeL15 and SeL60 treatments significantly deepened yolk color (*P* < 0.05). However, no significant effects were observed for egg shape index, eggshell thickness, eggshell breaking strength, or albumen height (*P* > 0.05).

**Table 5:** Effect of dietary Se-enriched lactobacilli on egg quality of laying hens^1^

Item	Experimental treatment^2^					SEM	*P* value
	SeD	SeY15	SeL15	SeL30	SeL60		
Egg shape index							
Week 0	1.40	1.38	1.38	1.39	1.40	0.006	0.587
Week 6	1.39	1.39	1.38	1.39	1.39	0.005	0.938
Week 12	1.33	1.35	1.34	1.35	1.35	0.004	0.263
Eggshell thickness, × 0.01 mm							
Week 0	36.74	37.80	37.99	39.60	36.89	0.690	0.726
Week 6	37.37	37.67	36.57	36.11	37.39	0.489	0.859
Week 12	37.89	37.40	37.78	37.33	37.64	0.110	0.459
Eggshell breaking strength, N							
Week 0	41.25	40.68	40.75	41.33	41.87	0.840	0.993
Week 6	41.42	40.37	41.39	40.80	41.46	0.628	0.980
Week 12	42.13	43.79	42.22	39.72	42.35	0.604	0.331
Albumen height, mm							
Week 0	8.35	7.97	7.68	8.17	8.14	0.132	0.589
Week 6	8.19	8.75	8.37	8.51	8.77	0.099	0.285
Week 12	7.65	8.50	8.14	8.33	8.11	0.114	0.172
Haugh unit							
Week 0	91.55	88.32	86.61	90.16	89.89	0.876	0.466
Week 6	89.70	93.06	92.98	91.52	89.24	0.541	0.054
Week 12	86.21^b^	91.68^a^	88.82^ab^	89.78^a^	88.82^ab^	0.569	0.032
Yolk color							
Week 0	4.92	5.06	5.17	5.14	4.95	0.084	0.859
Week 6	5.14	4.75	5.20	4.81	4.75	0.102	0.468
Week 12	4.86^c^	5.65^a^	5.33^ab^	5.06^bc^	5.39^ab^	0.076	0.004

^a,b^Within a row, means with no common superscript differ significantly (*P* < 0.05)

^1^Means were calculated using 6 replicates (6 eggs/replicate) per treatment

^2^*SeD* The basal diet without Se supplementation, *SeY15* The diet supplemented with 1.5 mg/kg Se from Se-enriched yeast, *SeL15*, *SeL30* or *SeL60* The diets supplemented with 1.5, 3.0 or 6.0 mg/kg Se from Se-enriched lactobacilli

### Se content in egg and thigh muscle

As presented in Table 6, dietary SeY and SeL extremely significantly improved the Se content in both eggs and thigh muscle at the conclusion of the trial, compared to the SeD group (*P* < 0.001). In the edible portion of the eggs, the Se concentrations were 700.33%, 205.75%, 264.53%, and 516.99% higher in the SeY15, SeL15, SeL30, and SeL60 groups, respectively, than in the SeD group (*P* < 0.001). Furthermore, the Se levels in the albumen and yolk presented 1,338.17%, 146.52%, 221.67% and 593.24% higher (*P* < 0.001), and 426.86%, 219.65%, 297.17% and 477.43% higher (*P* < 0.001), respectively. Regarding thigh muscle Se content, birds fed with SeL15 and SeL60 exhibited increases of 615.73%, 67.53% and 204.84% compared to the SeD group (*P* < 0.001). Additionally, positive linear and quadratic correlations were observed between dietary Se levels from SeL and the Se concentrations in edible part (*R*^2^ = 0.934, *P* < 0.001; *R*^2^ = 0.940, *P* < 0.001), albumen (*R*^2^ = 0.867, *P* < 0.001; *R*^2^ = 0.878, *P* < 0.001), and yolk (*R*^2^ = 0.925, *P* < 0.001; *R*^2^ = 0.966, *P* < 0.001), respectively.

**Table 6:** Effects of dietary Se-enriched lactobacilli on the concentration of Se in whole liquid egg, albumen, yolk and thigh muscle of laying hens^1^

Item	Experimental treatment^2^					SEM	*P* value^3^		
	SeD	SeY15	SeL15	SeL30	SeL60		ANOVA	Linear	Quadratic
Egg Se concentration, μg/100 g									
In edible part	12.01^d^	96.12^a^	36.72^c^	43.78^c^	74.10^b^	5.603	< 0.001	< 0.001	< 0.001
In albumen	5.03^d^	72.34^a^	12.40^c^	16.18^c^	34.87^b^	4.605	< 0.001	< 0.001	< 0.001
In yolk	30.08^d^	158.49^a^	96.15^c^	119.47^b^	173.69^a^	9.693	< 0.001	< 0.001	< 0.001
Thigh Se concentration, μg/100 g									
In thigh muscle	11.58^d^	82.84^a^	19.40^c^	—	35.30^b^	5.166	< 0.001	—	—

^a−d^Within a row, means with no common superscript differ significantly (*P* < 0.05)

^1^Means of egg Se concentration were calculated using 6 replicates (3 eggs/replicate) per treatment, while the mean of thigh muscle Se was calculated using 8 replicates per treatment (thigh muscle samples were only collected from SeD, SeY15, SeL15 and SeL60 groups)

^2^*SeD* The basal diet without Se supplementation, *SeY15* The diet supplemented with 1.5 mg/kg Se from Se-enriched yeast, *SeL15*, *SeL30* or *SeL60* The diets supplemented with 1.5, 3.0 or 6.0 mg/kg Se from Se-enriched lactobacilli

^3^The differences among all treatments were analyzed using one-way ANOVA, while the linear and quadratic effects were conducted using regression analysis among SeD, SeL15, SeL30 and SeL60 groups

### Serum biochemistry and liver histomorphology

**Fig. 1:**
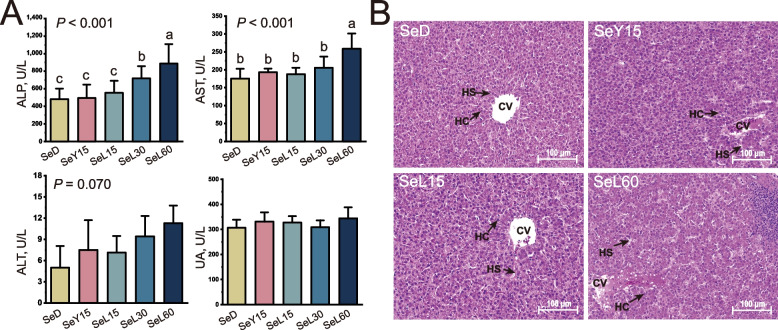
Effect of dietary Se-enriched lactobacilli on serum biochemistry and liver histomorphology of laying hens. **A** Concentration of serum alkaline phosphatase (ALP), aspartate transaminase (AST), alanine transaminase (ALT) and uric acid (UA). Data are mean ± SD (*n* = 8). Bars with no common letters differ significantly (*P* < 0.05). **B** Hematoxylin and erosion (H&E) staining of liver sections, scale bar: 100 μm. *CV* Central vein, *HC* Hepatic cell, *HS* Hepatic sinusoid, *SeD* The basal diet without Se supplementation, *SeY15* The diet supplemented with 1.5 mg/kg Se from Se-enriched yeast, *SeL15*, *SeL30* or *SeL60* The diets supplemented with 1.5, 3.0 or 6.0 mg/kg Se from Se-enriched lactobacilli

Serum ALP levels were significantly elevated in the SeL30 and SeL60 groups, while serum AST levels increased in the SeL60 group when compared to the SeD, SeY15, and SeL15 groups (*P *< 0.05; Fig. 1A). Liver sections from the SeD, SeY15 and SeL15 groups showed normal liver architecture (Fig. 1B). In contrast, SeL60 treatment caused marked cellular swelling with indistinct boundary and inflammatory cell infiltration.

### Antioxidant capacity assessment

Table 7 presents the influence of SeY and SeL on the serum antioxidant indices in hens. The dietary treatments with SeY and SeL extremely significantly improved serum GSH-Px activity (*P *< 0.001) and decreased MDA content (*P* < 0.001). Meanwhile, the activities of serum SOD, CAT, and T-AOC were also significantly elevated by the SeL15, SeL30, and/or SeY15 or SeL60 treatments (*P* < 0.05).

**Table 7:** Effect of dietary Se-enriched lactobacilli on the serum antioxidant indices of laying hens^1^

Item^2^	Experimental treatment					SEM	*P* value
	SeD	SeY15	SeL15	SeL30	SeL60		
T-AOC, U/mL	4.20^b^	5.45^a^	5.49^a^	5.46^a^	4.26^b^	0.163	0.003
CAT, U/mL	3.94^b^	4.29^ab^	4.96^a^	4.96^a^	4.45^ab^	0.113	0.009
SOD, U/mL	219.68^c^	295.62^c^	558.46^b^	579.49^b^	1,134.87^a^	57.600	< 0.001
GSH-Px, U/mL	417.32^d^	791.81^a^	588.53^c^	742.78^b^	623.17^c^	21.830	< 0.001
MDA, nmol/mL	66.44^a^	41.38^b^	46.94^b^	40.25^b^	40.88^b^	2.143	< 0.001

^a−d^ Within a row, means with no common superscript differ significantly (*P* < 0.05)

^1^ Means were calculated using 8 replicates per treatment

^2^ *SeD* The basal diet without Se supplementation, *SeY15* The diet supplemented with 1.5 mg/kg Se from Se-enriched yeast, *SeL15*, *SeL30* or *SeL60* The diets supplemented with 1.5, 3.0 or 6.0 mg/kg Se from Se-enriched lactobacilli. *T-AOC* Total antioxidant capacity, *CAT* Catalase, *GSH-Px* Glutathione peroxidase, *SOD* Superoxide dismutase, *MDA* Malondialdehyde

**Fig. 2:**
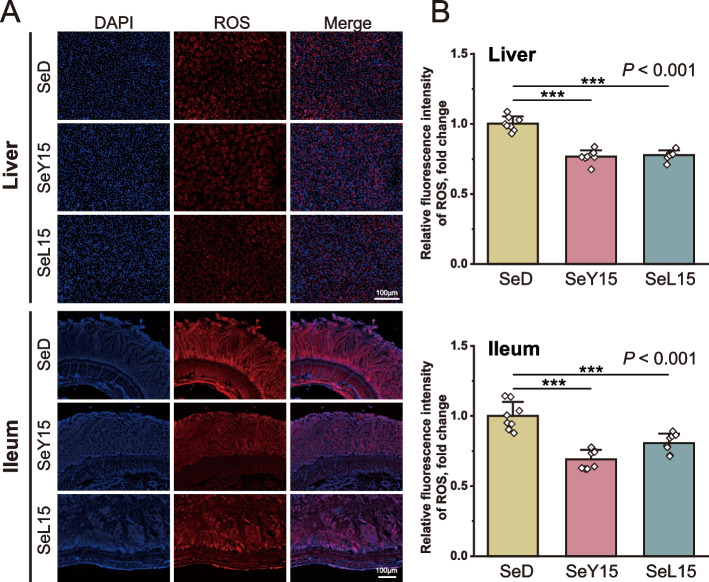
Effect of dietary Se-enriched yeast or lactobacilli on ROS levels of liver and ileum in laying hens. **A** Representative fluorescence images of liver and ileum frozen sections stained with DHE and DAPI, scale bar: 100 μm. **B** Relative fluorescence intensity of ROS in liver and ileum of layers. Data are mean ± SD (standard deviation) (*n* = 8), ^***^P < 0.001. *SeD* The basal diet without Se supplementation, *SeY15* or *SeL15* The diet supplemented with 1.5 mg/kg Se from Se-enriched yeast or Se-enriched lactobacilli

**Fig. 3:**
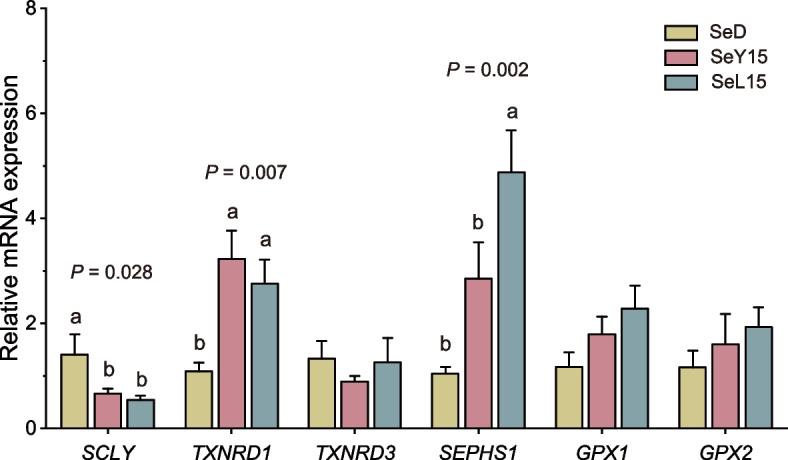
Effect of dietary Se-enriched yeast or lactobacilli on the relative mRNA expression of hepatic selenoprotein in laying hens. Data are mean ± SE (standard error) (*n* = 8). ^a,b^Bars with no common letter indicate statistical differences among 3 treatments (*P* < 0.05). *SeD* The basal diet without Se supplementation, *SeY15* or *SeL15* The diet supplemented with 1.5 mg/kg Se from Se-enriched yeast or Se-enriched lactobacilli

ROS concentration in the liver and ileum was detected using DHE fluorescence staining (Fig. 2). In comparison to the SeD group, both dietary SeY15 and SeL15 significantly decreased ROS levels in liver and ileum tissues (*P* < 0.001). Furthermore, treatments with SeY15 and SeL15 markedly up-regulated the mRNA expression of *TXNRD1* in the liver while down-regulating *SCLY* expression (*P *< 0.05; Fig. 3). SeL15 treatment also resulted in a significant up-regulation of *SEPHS1* mRNA expression in the liver when compared to either the SeD or SeY15 groups (*P* < 0.05).

### Ileal morphology and microbiota

**Fig. 4:**
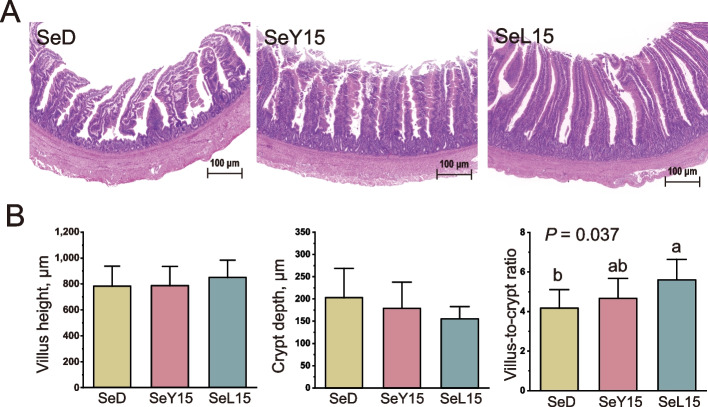
Effect of dietary Se-enriched yeast or lactobacilli on ileal morphology of laying hens. **A** Hematoxylin and erosion (H&E) staining of ileum sections, scale bar: 100 μm. **B** Ileal morphological parameters inclusive of villus height, crypt depth and villus height-to-crypt depth ratio. Data are mean ± SD (*n* = 8). ^a,b^Bars with no common letters differ significantly (*P* < 0.05). *SeD* The basal diet without Se supplementation, *SeY15* or *SeL15* The diet supplemented with 1.5 mg/kg Se from Se-enriched yeast or Se-enriched lactobacilli

As shown in Fig. 4, no significant differences were detected in VH or CD of the ileum among the SeD, SeY15 and SeL15 groups (*P* > 0.05). However, treatment with SeL15 indeed improved the ileal VH/CD compared to the SeD group (*P* < 0.05).

**Fig. 5:**
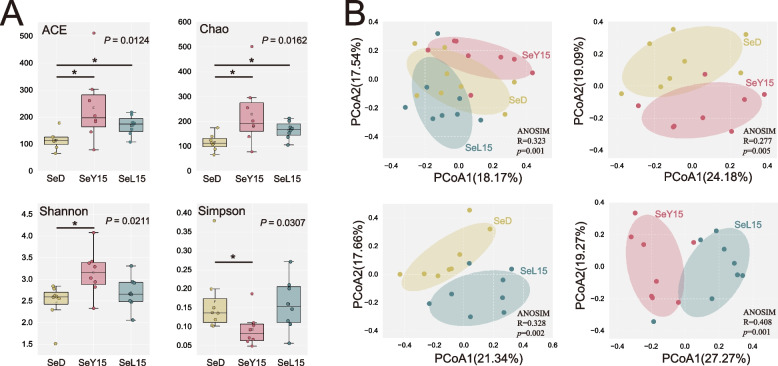
Effect of dietary Se-enriched yeast or lactobacilli on ileal bacterial diversity (α and β) in laying hens (*n* = 8). **A** Alpha diversity inclusive of Ace, Chao, Shannon and Simpson indices. **B** Beta diversity: principal coordinate analysis (PCoA) based on Bray-Curtis distances. The differentiation of microbial structure among treatments was statistically tested by analysis of similarity (ANOSIM). *SeD* The basal diet without Se supplementation, *SeY15* or *SeL15* The diet supplemented with 1.5 mg/kg Se from Se-enriched yeast or Se-enriched lactobacilli. ^*^P < 0.05

Alpha diversity of the ileal microbiota was assessed using the Ace, Chao, Shannon and Simpson metrics (Fig. 5A). Both SeY15 and SeL15 significantly raised Ace and Chao indices of richness (*P* < 0.05), while SeY15 also improved Shannon index and lowered Simpson index (*P* < 0.05). PCoA plots (Fig. 5B) revealed that the ileal microbial communities were markedly altered by the SeY15 (*P* = 0.005, ANOSIM) and SeL15 (*P* = 0.002, ANOSIM) compared to the SeD group. Notably, a significant separation was also observed between the SeY15 and SeL15 groups (*P* = 0.001, ANOSIM).

**Fig. 6:**
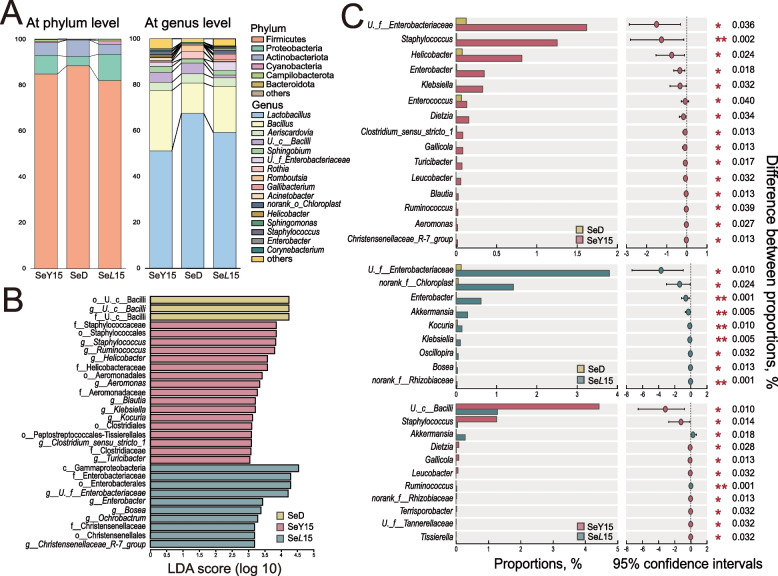
Effect of dietary Se-enriched yeast or lactobacilli on ileal bacterial composition in laying hens (*n* = 8). **A** Microbial composition in layers’ ileum at the phylum and genus levels under three treatments. **B** Linear discriminant effect size of ileal microbiota (LDA > 3.0; *P* < 0.05). **C** Differences in relative abundance of ileal microbiota at genus level in laying hens from different groups. SeD The basal diet without Se supplementation, SeY15 or SeL15 The diet supplemented with 1.5 mg/kg Se from Se-enriched yeast or Se-enriched lactobacilli

As illustrated in Fig. 6A, the dominant phylum in hens’ ileum was Firmicutes, contributing > 80% to the whole community. Both SeY15 and SeL15 treatments lowered the relative abundance of Firmicutes and Actinobacteriota but increased Proteobacteria. *Lactobacillus* and *Bacillus*, belonging to Firmicutes, were the most abundant genera in the ileum, with *Bacillus* being more abundant, and *Lactobacillus* being less abundant in SeY15 and SeL15 groups than those in the SeD group. LEfSe analysis (*P* < 0.05; LDA > 3.0) was employed to identify the significant differentially abundant taxa in different groups (Fig. 6B). Only one unclassified order/family/genus member belonging to class Bacilli was differentially enriched in the SeD group. For SeY15 treatment, LEfSe highlights substantial bacterial members enriched in the ileum, including orders Staphylococcales (Staphylococcaceae, *Staphylococcus*), Clostridiales (Clostridiaceae, *Clostridium*_*sensu*_*stricto*_*1*), Aeromonadales (Aeromonadaceae, *Aeromonas*), family Helicobacteraceae (*Helicobacter*) and genera *Turicibacter*, *Ruminococcus*, *Klebsiella*, *Blautia*, etc. Birds in the SeL15 group exhibited enriched abundances of class Gammaproteobacteria and its derivatives (Enterobacterales, *Enterobacteriaceae*, *Enterobacter*), order Christensenellales and its derivatives (*Christensenellaceae*, *Christensenellaceae_R-7_group*). Wilcoxon rank-sum test performed between SeD and SeY15 or SeL15 at genus level supported the above results (Fig. 6C). In addition, SeY15 also triggered higher abundances of *Enterobacter*, *Enterococcus*, *Dietzia*, *Christensenellaceae_R-7_group*, etc., while SeL15 increased abundances of *Akkermansia* and *Oscillospira* when compared with SeD group (*P* < 0.05). Notably, SeL15 also lowered abundances of *Staphylococcus*, *Dietzia*, *Gallicola*, *Tissierella* but elevated *Akkermansia* and *Ruminococcu*s in comparison with SeY15 group (*P* < 0.05).

## Discussion

This study was conducted to compare the effects of SeL and SeY on production performance, egg Se concentration, antioxidant capacity, and ileal microbiota in laying hens. The results demonstrated that both SeL and SeY positively affected production, albeit with distinct advantages: SeY excelled in enhancing Se deposition in eggs and tissues, while SeL showed greater efficacy in modulating gut microbiota and improving antioxidant defenses. These findings provide new insights into the roles of Se-enriched probiotics in poultry nutrition, particularly regarding their combined effects on Se bioavailability and gut health.

Our study revealed that both SeY and SeL, when administered at a moderate dose of 1.5 mg Se/kg feed, improved the feed-to-egg ratio and hen-day egg production during the later stages of the trial. These results align with previous findings that demonstrated the benefits of organic Se sources, such as Se-enriched yeast [11] and Se-enriched *Stenotrophomonas maltophilia* [36], in enhancing production performance. Specifically, the SeY15 and SeL15 groups exhibited superior laying performance and feed efficiency compared to the SeD and SeL60 groups. Supplementation with Se-Met (0.15 and 0.3 mg Se/kg diet) resulted in increased egg production relative to sodium selenite in a non-dose-dependent manner [3]. This improvement in production efficiency may be attributed to the high bioavailability of organic Se, which enhances the metabolic function of antioxidant enzymes and supports overall health in laying hens. However, some studies have indicated that neither SeY nor Se-Met had a significant impact on laying performance within the tested dosage range [10, 12]. The discrepancies in findings may be attributed to various factors, including the additional benefits of the Se source (e.g., probiotics properties), duration of feeding, and environmental stressors [3, 25]. While Se-enriched diets may promote metabolic functions and overall health for increased performance, it becomes expedient to assess if the nutrient absorption had an impact on egg quality which is also an economic trait for the poultry industry.

Egg freshness is a critical parameter highly valued by consumers, with HU and albumen height serving as important indicators of internal quality [37]. In the current study, supplementation with either SeY or SeL, significantly enhanced HU values, which are of great importance to the food processing industry. This finding corroborates previous research indicating that various forms of organic Se, including SeY, increase HU compared to inorganic selenite and non-supplemented groups [24, 36, 38]. Additionally, organic Se sources such as Se-enriched probiotics [25], selenized glucose [13], and Se-chitosan [24] have been reported to extend the shelf life of eggs by slowing the decline in HU values. Thus, future research may involve investigating the effect of SeL on the internal quality of egg traits during storage, using the optimal dosage validated in this trial. Egg yolk color is another critical criterion for consumers, as darker yolks are generally perceived as more appealing [39]. Our study demonstrated that both SeY and SeL significantly enhanced yolk color. Similarly, Muhammad et al. [40] found that supplementation with a Se-enriched probiotic (*Stenotrophomonas maltophilia*-ADS18) led to significantly higher yolk color scores, as well as increased yellowness (b*) and Chroma (C*) values. Also, Wang et al. [41] reported that hens receiving Se-enriched yeast showed darker yolks compared to those fed sodium selenite, highlighting Se source as a contributory factor to yolk color. Xanthophylls, or oxycarotenoids, are the primary pigments responsible for yolk coloration. These pigments can degrade when exposed to oxidative stress, leading to diminished yolk intensity. The antioxidant properties of Se may contribute to the stabilization of yolk color by preventing the oxidation of xanthophyll pigments [42].

Both improved albumen quality and enhanced yolk color can be attributed to the increased antioxidant capacity conferred by elevated Se levels in eggs. The activity of GSH-Px was found to play a critical role in protecting eggs from oxidative damage caused by free radicals [12] and has been shown to prevent the decline in egg HU [25]. Furthermore, yolk color has been positively associated with the activity of antioxidant enzymes (GSH-Px, SOD, and CAT) and yolk Se concentration, while showing a negative relationship to MDA content, a marker of lipid peroxidation [24]. Se-enriched probiotic has been found to enhance the antioxidant capacity of egg yolk, preserving the oxidative stability [40]. This substantiates our findings that the significant increase in GSH-Px activity and the decrease in MDA content, may account for the improved egg quality traits. Overall, these findings highlight the beneficial effects of organic Se, particularly SeY and SeL, in improving egg quality via enhanced antioxidant defense. Since Se supplementation is expected to enhance the nutritional profile, it is valuable to measure Se deposition in eggs and muscle tissue.

As expected, Se concentrations in whole egg, albumen, and yolk, as well as the thigh muscle increased with Se supplementation from both SeY and SeL, compared to the control. These results align with previous studies [11, 12, 15, 25, 43]. Organic Se has been confirmed to be more effective in enhancing Se content in meat and eggs compared to its inorganic counterpart [3, 26, 44]. In a study by Utterback et al. [44], there was a 4.8-fold increase in egg Se concentration after supplementation with SeY, compared to a 2.8-fold increase with sodium selenite over a 56-day period. This phenomenon can be explained by the theory of adaptive evolution, which posits that organic Se closely resembles the natural form of Se present in the raw materials of hens diet. Se-Met has been identified as the predominant seleno-compound in both SeY (60%–80%, as reported by Surai and Fisinin [45]) and SeL (68.64%, as determined according to the Chinese industry standard GH/T 1135–2017 'Selenium-enriched agricultural products' in this trial). The Se-Met mimics the natural methionine which is more readily absorbed and incorporated into egg proteins and tissues, thus, replacing methionine and enhancing its bioavailability compared to other sources [46]. The higher efficacy of SeY compared to SeL in promoting Se deposition in whole egg, albumen, yolk, and thigh muscle, could be explained by the fact that SeL enhances Se utilization by regulating physiological processes, which aids biofortification in laying hens. This hypothesis is partially supported by the observed improvements in ileal morphology and up-regulation of liver *SEPHS1* gene expression, though further investigation is needed to elucidate the precise mechanisms involved. A particularly noteworthy finding among the SeL groups was that Se deposition efficiency in egg components, when calculated based on Se intake, was highest at a dietary Se concentration of 1.5 mg/kg, followed by moderate efficiency at 3.0 mg/kg and the lowest efficiency at 6.0 mg/kg. These results suggest that Se utilization in laying hens is more efficient at lower dietary Se concentrations, aligning with previous findings by Yoon et al. [47] and Delezie et al. [15]. These could have important implications for optimizing egg production and nutritional value.

One critical concern when supplementing diets with high doses of organic Se to produce Se-enriched eggs is the potential toxicity to hens, which may adversely affect their health and productivity. The National Research Council [48] has established the maximum tolerable Se level for poultry at 3 mg/kg of dry matter (DM) feed. Similarly, Lu et al. [10] reported that up to 3 mg/kg of organic Se from SeY could be safely included in layer diets over a 12-week period without causing adverse effects. This finding is supported by the European Food Safety Authority [20], which concluded that SeY could be used in laying hen diets at levels up to 5.7 mg Se/kg of complete feed. However, an earlier study [49] indicated that higher Se dosages, such as 7 mg/kg, could lead to a reduction in the laying rate. In the current study, 6.0 mg Se/kg diet from SeL led to declines in production performance and feed intake, suggesting potential Se toxicity at this dosage. In addition, toxic effects were evidenced by the alterations in serum biochemical indices and liver histopathological examinations, which showed elevated serum levels of ALP and AST, alongside histopathological observations of liver cell swelling and inflammatory cell infiltration. Similarly, previous studies have reported toxic effects, where elevated SeY levels (20 or 30 mg/kg feed) induced pathological alterations in the liver and kidneys of birds [50]. The observed toxicity at these elevated levels may be attributed to the disruption of protein integrity, as high Se concentrations can affect thiol affinity, which is essential for DNA repair [51]. Additionally, Se toxicity can catalyze hydrosulfide oxidation, inhibiting protein synthesis [52], and causing significant liver damage. Taken together, an appropriate SeL concentration (1.5 mg Se/kg feed) can promote production effects comparable to SeY without toxicity. However, a high SeL level (6.0 mg Se/kg feed) results in Se toxicity, manifesting in decreased egg production and feed intake, along with disrupted metabolic function and hepatocyte degeneration. Given selenium’s documented antioxidant properties, we subsequently evaluate its impact on the hens' systemic antioxidant defense by measuring serum antioxidant capacity.

Animal well-being can be enhanced by improving antioxidant capacity. Key components of physiological antioxidant systems include endogenous antioxidant enzymes, such as GSH-Px, SOD, and CAT, as well as non-enzymatic constituents like lactoferrin, carotene, vitamin C, and glutathione [53]. GSH-Px, an intracellular enzyme that relies on Se, plays a crucial role in reducing hydrogen peroxide and organic peroxides to water and corresponding alcohols, thus preventing the formation of harmful free radicals [54]. SOD catalyzes the dismutation of superoxide anions into hydrogen peroxide and oxygen [55], while CAT converts hydrogen peroxide into water and oxygen, providing protection against oxidative damage [26, 56]. Additionally, MDA serves as a biomarker for lipid peroxidation levels, indicating the extent of oxidative stress [57]. In the present study, Se supplementation from either SeY or SeL, significantly increased the activity of T-AOC and GSH-Px, while simultaneously reducing MDA concentration in the serum. These findings align with previous studies indicating that organic Se possesses a more potent antioxidant effect compared to inorganic forms and non-supplemented treatments, which can be attributed to its superior bioavailability [14, 26]. Additionally, SeL supplementation also enhanced the activities of serum SOD and CAT, although these enzymes are not directly Se-dependent. The inclusion of Se in the form of SeL may influence their activity indirectly, possibly through the modulation of thyroid hormone levels [26]. In addition to the antioxidant capacity of the serum, elevated concentrations of ROS in both the liver and ileum can compromise productivity and health of laying hens. In the liver, ROS lead to lipid peroxidation, hepatocyte apoptosis, and chronic inflammation, impairing the organ's detoxification and metabolic functions. This results in reduced laying performance and compromised egg quality [58, 59]. Similarly, in the ileum, ROS weakens the intestinal barrier, disrupts gut microbiota, and damages villi, leading to poor nutrient absorption and increased inflammation [60]. These effects contribute to nutritional deficiencies and an overall reduction in the health of hens. The findings revealed a significant reduction in ROS levels in the liver and ileum tissues of hens fed either SeY or SeL compared to the control group. SeY has been observed to reduce ROS activity in broilers exposed to heat stress [61], which supports our findings. The molecular mechanisms underlying the significant reduction of ROS in tissues were further investigated using liver tissue to assess the expression of selenoproteins.

It is hypothesized that the reduction of accumulated ROS is associated with the action of selenoproteins, such as glutathione peroxidase 1 (GPX1), TXNRD1, TXNRD3, and SEPHS1, following dietary Se supplementation [62, 63]. In our study, both SeY and SeL up-regulated the mRNA expression of *TXNRD1* in the liver, while simultaneously down-regulating the expression of *SCLY*. Notably, SeL supplementation also led to an increased mRNA expression of *SEPHS1* in the liver. TXNRD1 functions by reducing oxidized thioredoxin, along with other disulfide-containing molecules or proteins, thereby playing a crucial role in maintaining cellular redox homeostasis [64]. Consistent with our findings, Wang et al. [65] reported that Se supplementation reduced ROS levels and enhanced the viability and proliferation of SH-SY5Y cells through increased *TXNRD1* expression. While, SCLY is an enzyme involved in the decomposition of selenocysteine to elemental Se for selenoprotein biosynthesis [66], a process that appears to have been suppressed by organic Se supplementation in this trial. Probably because, organic Se provides a more direct and bioavailable source of Se for incorporation into proteins and selenoproteins, reducing the need for the SCLY-mediated breakdown and recycling of selenocysteine. Moreover, SEPHS1 is an enzyme responsible for catalyzing the synthesis of selenophosphate from selenide in an ATP-dependent manner [67]. Studies demonstrated that *SEPHS1*-knockout cells accumulate excessive ROS and exhibit significant changes in the expression of genes involved in the ROS pathway [68, 69]. This suggests that the up-regulation of these genes is one of the mechanisms by which organic Se mitigates oxidative stress. Collectively, our findings indicate that SeL and SeY improved the antioxidant capacity of laying hens, as demonstrated by increased serum antioxidant enzyme activity and reduced ROS levels in both the liver and ileum. These effects may be mediated by altered expression of key selenoproteins.

Investigating the structural integrity of the ileum reveals how Se supplementation affects nutrient absorption and gut health. The addition of SeL at 1.5 mg Se/kg feed significantly improved the ileal villus-to-crypt ratio by the end of the trial, whereas the same level of Se from SeY did not affect ileal morphology compared to the SeD group. This suggests that the presence of lactobacilli, rather than organic Se alone, exerts a more beneficial impact on gut morphology. Numerous studies have highlighted the crucial role of lactobacilli in promoting intestinal health and function [70, 71], primarily through its interaction with the gut microbiota. Moreover, dietary Se has been shown to influence gut microbiota in laying hens [72, 73], prompting further analysis of the ileal microbiota in this study, owing to the critical role of microbiota in nutrient metabolism and overall health.

Increased bacterial diversity generally reflects a more stable microbiota community, which benefits the host by preventing pathogen colonization and promoting overall health and performance [74]. Consistent with previous findings [75], both SeY and SeL supplementation (1.5 mg Se/kg feed) in our study increased the richness of the ileal microbiota, with SeY also improving microbial evenness. Beta diversity analysis further revealed significant alterations in microbial community structure due to SeY and SeL, with distinct differences also observed between the SeY15 and SeL15 groups, suggesting the involvement of different microbial mechanisms in response to these treatments. Analysis of microbial composition revealed that both SeY and SeL triggered an increase in the *Bacillus* genus, a probiotic known for its production of digestive enzymes such as protease, amylase and lipase [76]. Further microbial analysis using LEfSe and the Wilcoxon rank-sum test identified several taxa that underwent significant changes. Birds receiving SeY supplementation exhibited increases in beneficial bacteria, including *Turicibacter*, *Blautia*, and *Clostridium_sensu_stricto_1*, but also in harmful pathogens like *Staphylococcus*, *Enterobacter*, and *Helicobacter*. *Turicibacter* aids in lipid metabolism via bile acid modification and has been noted to rise with SeY treatment in layers [14]. *Blautia* supports gut health by producing organic acids and vitamins [77], while *Clostridium_sensu_stricto_1*, a potential probiotic, enhances the intestinal mucus barrier by producing short-chain fatty acids from mucus-derived sugars, thereby preventing pathogen adhesion [78]. However, the increase in pathogens such as *Staphylococcus, Enterobacter*, and *Helicobacter* is concerning, as these bacteria can negatively impact animal health and production, leading to economic losses [79]. In contrast, SeL supplementation tended to boost beneficial bacteria like *Blautia, Christensenellaceae_R-7_*group, *Akkermansia*, and *Oscillospira*, which are known for their roles in gut immune regulation and the improvement of intestinal integrity [80, 81]. The positive correlation between these bacteria and improved ileal morphology in laying hens aligns with our findings [82]. Moreover, when compared with SeY group, SeL supplementation reduced the abundance of pathogens like *Staphylococcus*, *Dietzia*, *Gallicola*, and *Tissierella*, while significantly increasing beneficial taxa like *Akkermansia* and *Ruminococcus*. *Akkermansi*a spp. play crucial roles in regulating intestinal immunity, enhancing the integrity of intestinal epithelial cells, and increasing the thickness of the mucus layer [80, 82]. *Ruminococcus* has been shown to be closely associated with the lipid metabolism of animals via the bile-acid-TGR5 axis, in addition to fortifying the intestinal barrier [83, 84]. These findings suggest that SeL may exert more favorable influence on gut microbiota modulation compare to SeY, potentially improving gut morphology and reducing pathogen load.

## Conclusion

Our study demonstrated that Se derived from SeL can achieve similar improvements in antioxidant capacity, production performance, and egg quality as Se from SeY. However, SeY proved to be more efficient in Se deposition in both eggs and tissues, while SeL showed superior effects on gut microbiota modulation. These findings suggest that SeL has significant potential as an effective feed additive, combining the antioxidant benefits of organic Se with the probiotic advantages for gut health, ultimately enhancing the overall performance of laying hens. Nonetheless, it is crucial to manage SeL supplementation carefully, as dosages exceeding 3.0 mg Se/kg may pose toxic risks, according to the results of this trial.

## Data Availability

The sequencing datasets are available in the Sequence Read Archive of National Center for Biotechnology Information (accession numbers: PRJNA1120179).

## Abbreviations

ANOSIM: Analysis of similarity
ALP: Alkaline phosphatase
AST: Aspartate transaminase
CAT: Catalase
CD: Crypt depth
GPX1: Glutathione peroxidase 1
GSH-Px: Glutathione peroxidase
LDA: Linear discriminant analysis
MDA: Malondialdehyde
PCoA: Principal coordinate analysis
ROS: Reactive oxygen species
SCLY: Selenocysteine lyase
Se-Met: Selenomethionine
SEPHS1: Selenophosphate synthetase 1
SeL: Selenium-enriched lactobacilli
SeY: Selenium-enriched yeast
SOD: Superoxide dismutase
T-AOC: Total antioxidant capacity
TXNRD1: Thioredoxin reductase 1
VH: Villus height
